# Supplementary material for: Association of Organizational Behavior with Work Engagement and Work-Home Conflicts of Physician in China
Source: Int J Environ Res Public Health. 2021 May 19;18(10):5405. doi: 10.3390/ijerph18105405 (PMC8158697; doi:10.3390/ijerph18105405)
Supplement: Supplementary file 1 [file ijerph-18-05405-s001.zip › ijerph-1121861-supplementary.pdf]

Supplementary Online Content

Association of Organizational Behavior with Work Engagement and Work-home Conflicts of Physician in China

Figure S1. Flowchart for recruitment and response rates of the participants

Table S1. Adjusted sex and age of the sample according to 2015 National Statistical Yearbook

Table S2. Reliability test: Work Engagement and Work-home Conflicts of Physician

Table S3. Correlations among index of Physician' Work Engagement and Work-home Conflicts of Physician

Table S4. Organizational behavior of Physician

Table S5.Work Engagement and Work-home Conflicts of Physician

Table S6. eTable6. Socio-demographic characteristics, hospital and departmental characteristics, family support, and patient behavior with work engagement of physician

Table S7. eTable6. Socio-demographic characteristics, hospital and departmental characteristics, family support, and patient behavior with Work-home Conflicts of physician

Table S8. Descriptive statistics for Organizational Behavior with Work Engagement of Physician

Table S9. Descriptive statistics for Organizational Behavior with Work-home Conflicts of Physician

Table S10. Multivariable Logistic Regression Results for Correlates of Work Engagement and Work-home Conflicts of Physician-Sensitivity Analysis

Table S11. Multivariable Logistic Regression Results for Correlates of Work Engagement or Work-home Conflicts of Physician-Sensitivity Analysis

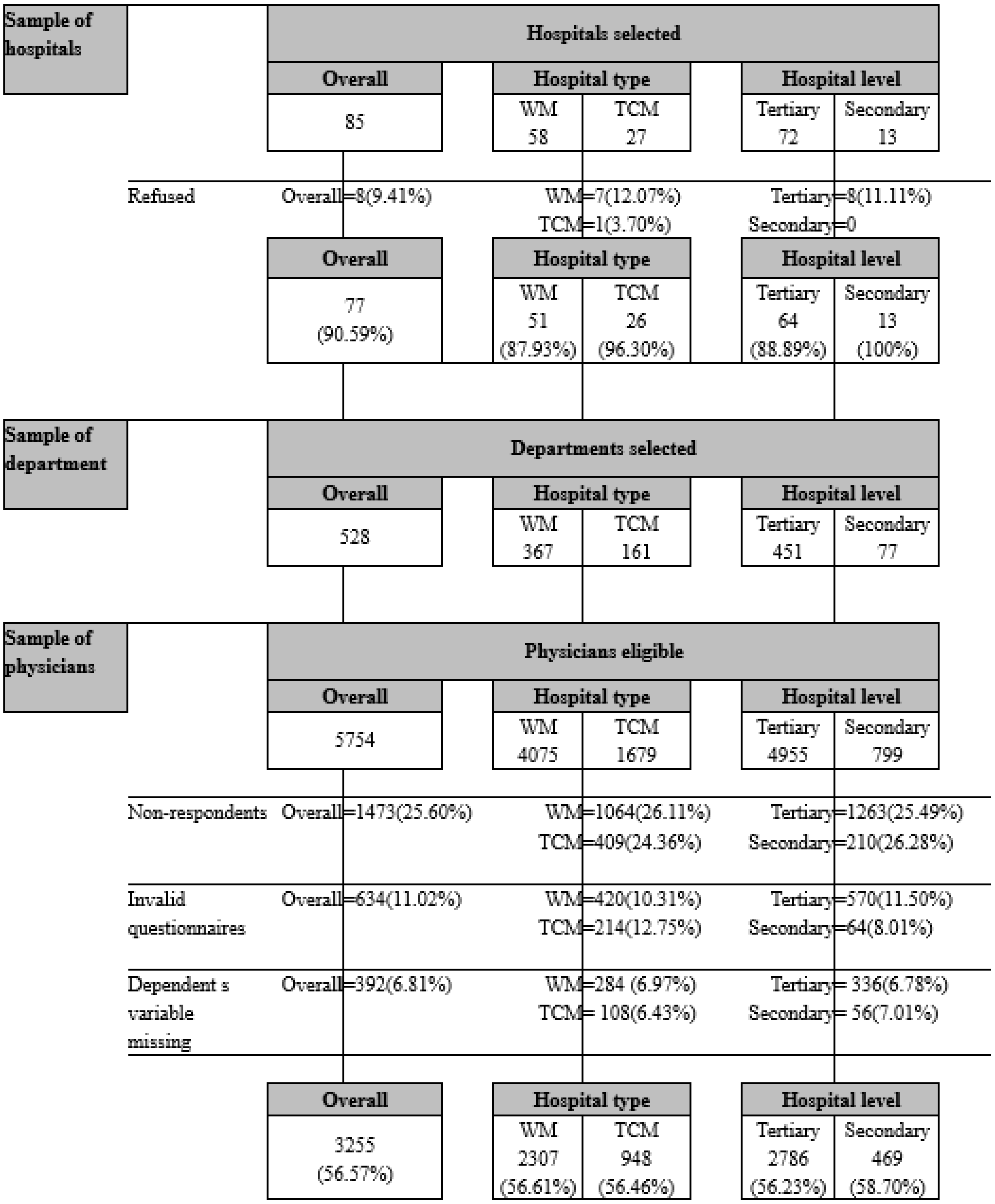

Figure S1. **Flowchart for recruitment and response rates of the participants.**

WM, Western Medicine. TCM, Traditional Chinese Medicine.

Table S1. Adjusted sex and age of the sample according to 2015 National Statistical Yearbook

Age	2015 National Statistical Yearbook		Sample		Weighting factor	
	Men（A）	Women（B）	Men（C）	Women（D）	Men（A/C）	Women（B/D）
≤34	16.41	12.59	30.29	25.02	0.54174570273003	0.50313769889841
35-44	19.64	15.06	19.17	9.98	1.02435463258786	1.50830981595092
≥45	20.55	15.75	10.84	4.69	1.89536016949153	3.36102941176471

Table S2. Reliability test: Work Engagement and Work-home Conflicts of Physician

Measuring scales	Items	Alpha
Work-home Conflicts：I1, I2, I3	3	0.632
Work engagement：C4, D1	2	0.813

Table S3 Correlations among index of Physician' Work Engagement and Work-home Conflicts of Physician

	Pride	Pleasure	Affecting care for family	Feeling guilty towards family	Receiving complaint from family
Pride	1				
Pleasure	0.685**	1			
Affecting care for family	-0.007	-0.048**	1		
Feeling guilty towards family	-0.197**	-0.213**	0.398**	1	
Receiving complaint t from family	-0.207**	-0.252**	0.243**	0.553**	1

Table S4. Organizational behavior of Physician

Variable description	N (%)	Recategorization
**Organizational fairness**		
Pay equity		
Very bad	906(27.88)	No
Somewhat bad	901(27.74)	No
Neutral	1186(36.50)	No
Somewhat good/	229(7.06)	Yes
Very good	27(0.83)	Yes
Task fairness		
Very bad	491(15.11)	No
Somewhat bad	699(21.51)	No
Neutral	1622(49.93)	No
Somewhat good/	385(11.86)	Yes
Very good	52(1.59)	Yes
**Leadership attention**		
Interests attention		
Very bad	1019(31.35)	No
Somewhat bad	772(23.76)	No
Neutral	1164(35.82)	No
Somewhat good/	243(7.48)	Yes
Very good	51(1.58)	Yes
Opinions attention		
Very bad	1083(33.30)	No
Somewhat bad	787(24.18)	No
Neutral	1069(32.88)	No
Somewhat good/	267(8.22)	Yes
Very good	46(1.42)	Yes
**Team interaction**		
Number of dinners with colleagues		
0 time	1440(44.40)	No
1 time	961(29.64)	No
2 times	444(13.70)	No
3 times	167(5.16)	Yes
≥4 times	230(7.10)	Yes
Number of clinical case meetings		
0 time	275(8.49)	No
1 time	488(15.08)	No
2 times	634(19.58)	No
3 times	512(15.82)	Yes
≥4 times	1329(41.03)	Yes

Table S5.Work Engagement and Work-home Conflicts of Physician

Variable description	N (%)	Recategorization
**Work engagement**		
Pride		
Very low	594(18.23)	No
Somewhat low	751(23.08)	No
Neutral	1339(41.12)	No
Somewhat high	478(14.69)	Yes
Very high	94(2.88)	Yes
Pleasure		
Very low	712(21.87)	No
Somewhat low	922(28.34)	No
Neutral	1115(34.25)	No
Somewhat high	432(13.27)	Yes
Very high	74(2.27)	Yes
**Work-home Conflicts because of work commitment**		
Affecting care for family		
Very low	408(12.53)	No
Somewhat low	422(12.96)	No
Neutral	500(15.37)	No
Somewhat high	869(26.69)	Yes
Very high	1056(32.44)	Yes
Feeling guilty towards family		
Very low	49(1.51)	No
Somewhat low	149(4.57)	No
Neutral	532(16.33)	No
Somewhat high	1184(36.37)	Yes
Very high	1342(41.23)	Yes
Receiving complaint from family		
Very low	141(4.32)	No
Somewhat low	440(13.52)	No
Neutral	951(29.21)	No
Somewhat high	976(29.99)	Yes
Very high	747(22.96)	Yes

Table S6. Socio-demographic characteristics, hospital and departmental characteristics, family support, and patient behavior with work engagement of physician

	Pride				Pleasure			
	Low	Neutral	High	*P* value	Low	Neutral	High	*P* value
**Socio-demographic characteristics**								
Sex								
Men	25.94	16.66	57.41	<0.001	47.91	35.43	16.66	0.002
Women	24.91	13.73	61.36		53.22	32.70	14.08	
Age, y								
≤34	25.53	16.42	58.05	<0.001	48.78	37.04	14.18	<0.001
35-44	26.53	13.58	59.89		56.79	30.70	12.51	
≥45	24.43	16.23	59.34		45.14	35.42	19.44	
Marital status								
Single/other	26.07	17.51	56.42	0.004	44.47	39.81	15.73	<0.001
Married	25.03	14.95	60.02		51.59	33.27	15.14	
Education level								
Undergraduate and below	25.19	16.44	58.37	<0.001	53.81	31.23	14.96	<0.001
Masters	25.42	13.99	60.58		49.43	35.99	14.59	
PhD	22.31	14.60	63.09		39.01	40.93	20.05	
Economic status								
Very bad	33.02	6.65	60.33	<0.001	73.63	19.71	6.65	<0.001
Somewhat bad	26.19	14.70	59.11		59.26	31.55	9.19	
Neutral	23.16	17.33	59.51		45.72	38.10	16.18	
Good	27.51	17.10	55.39		22.30	37.55	40.15	
Title								
Primary / other	26.51	17.00	56.48	<0.001	48.70	36.74	14.55	<0.001
Middle	25.47	15.10	59.43		57.71	29.96	12.33	
High	23.68	14.68	61.64		44.71	36.65	18.64	
**Hospital and Departmental characteristics**								
Hospital level								
Secondary	27.09	13.65	59.27	<0.001	55.31	30.61	14.08	0.010
Tertiary	25.21	15.70	59.10		49.28	34.89	15.84	
Hospital type								
Traditional Chinese medicine	25.88	17.59	56.53	0.025	48.45	35.40	16.15	0.033
Western medicine	25.31	14.50	60.19		50.89	33.80	15.31	
Academic status								
Nonteaching	25.98	14.54	59.49	<0.001	52.73	32.93	14.34	<0.001
Teaching	23.56	18.72	57.72		39.84	39.69	20.47	
Physician specialty								
Internal medicine	24.59	14.21	61.19	<0.001	52.90	31.90	15.20	<0.001
Surgery	26.50	16.71	56.79		47.16	36.90	15.94	
The ratio of physicians to beds								
<0.20	25.03	12.35	62.62	0.007	52.95	32.76	14.29	0.146
0.20-0.30	25.54	15.87	58.59		49.12	34.84	16.04	
≥0.30	25.88	17.45	56.67		49.12	34.90	15.98	
**Family support**								
Very low / Somewhat low	44.74	14.04	41.23	<0.001	64.91	23.68	11.40	<0.001
Neutral	26.78	25.39	47.83		54.53	36.59	8.89	
Somewhat high / Very high	24.38	13.22	62.40		48.54	34.26	17.21	
**Patient behavior**								
Patient trust								
Very low / Somewhat low	25.29	10.10	64.61	<0.001	66.91	25.96	7.13	<0.001
Neutral	26.07	17.91	56.02		41.44	41.78	16.78	
Somewhat high / Very high	24.45	24.18	51.37		23.01	34.25	42.74	
Unreasonable request from the patient								
Very low / Somewhat low	25.12	20.63	54.25	<0.001	40.55	36.83	22.61	<0.001
Neutral	24.05	16.24	59.71		44.50	39.69	15.80	
Somewhat high / Very high	27.04	9.53	63.43		65.12	26.25	8.63	

Table S7. Socio-demographic characteristics, hospital and departmental characteristics, family support, and patient behavior with Work-home Conflicts because of work commitment

	Affecting care for family				Feeling guilty towards family				Receiving complaint from family			
	Low	Neutral	High	*P* value	Low	Neutral	High	*P* value	Low	Neutral	High	*P* value
**Socio-demographic characteristics**												
Sex												
Men	25.94	16.66	57.41	0.132	6.41	17.37	76.22	0.092	16.56	29.21	54.23	0.013
Women	24.91	13.73	61.36		5.59	15.00	79.41		19.52	29.21	51.27	
Age, y												
≤34	25.53	16.42	58.05	0.759	6.35	18.52	75.13	0.004	19.79	30.90	49.31	0.001
35-44	26.53	13.58	59.89		6.12	13.93	79.95		14.82	29.46	55.72	
≥45	24.43	16.23	59.34		5.83	16.91	77.26		19.19	27.56	53.25	
Marital status												
Single/other	26.07	17.51	56.42	0.090	7.77	21.36	70.87	<0.001	20.93	31.59	47.48	<0.001
Married	25.03	14.95	60.02		5.56	15.37	79.07		17.16	28.62	54.21	
Education level												
Undergraduate and below	25.19	16.44	58.37	0.118	6.37	17.27	76.35	0.022	18.58	29.72	51.70	0.015
Masters	25.42	13.99	60.58		5.67	14.53	79.81		15.75	28.67	55.58	
PhD	22.31	14.60	63.09		6.87	17.03	76.10		21.15	29.40	49.45	
Economic status												
Very bad	33.02	6.65	60.33	0.436	5.46	7.13	87.41	<0.001	11.40	17.34	71.26	<0.001
Somewhat bad	26.19	14.70	59.11		4.90	15.47	79.63		16.06	27.68	56.27	
Neutral	23.16	17.33	59.51		5.68	17.97	76.35		18.39	32.00	49.61	
Good	27.51	17.10	55.39		12.64	20.82	66.54		28.62	30.86	40.52	
Title												
Primary / other	26.51	17.00	56.48	0.050	7.34	20.29	72.37	<0.001	20.43	30.22	49.35	0.014
Middle	25.47	15.10	59.43		5.73	15.33	78.94		16.10	30.32	53.58	
High	23.68	14.68	61.64		5.53	15.32	79.15		17.85	28.12	54.03	
**Hospital and Departmental characteristics**												
Hospital level												
Secondary	27.09	13.65	59.27	0.882	5.92	18.98	75.10	0.250	17.96	29.18	52.86	0.536
Tertiary	25.21	15.70	59.10		6.08	15.84	78.08		17.82	29.21	52.96	
Hospital type												
Traditional Chinese medicine	25.88	17.59	56.53	0.090	6.41	18.34	75.25	0.003	15.14	32.71	52.15	0.725
Western medicine	25.31	14.50	60.19		5.95	15.57	78.48		18.89	27.86	53.25	
Academic status												
Nonteaching	25.98	14.54	59.49	0.735	5.66	15.53	78.81	<0.001	17.51	28.87	53.61	0.047
Teaching	23.56	18.72	57.72		7.80	19.66	72.54		19.19	30.58	50.23	
Physician specialty												
Internal medicine	24.59	14.21	61.19	0.018	5.16	15.60	79.23	0.014	18.28	29.19	52.52	0.531
Surgery	26.50	16.71	56.79		7.11	17.17	75.72		17.30	29.24	53.46	
The ratio of physicians to beds												
<0.20	25.03	12.35	62.62	0.095	4.08	13.96	81.95	0.001	15.34	29.72	54.94	0.031
0.20-0.30	25.54	15.87	58.59		6.21	17.02	76.76		18.40	28.30	53.30	
≥0.30	25.88	17.45	56.67		7.74	17.63	74.63		19.43	29.93	50.64	
**Family support**												
Very low / Somewhat low	44.74	14.04	41.23	<0.001	28.70	15.65	55.65	<0.001	32.46	15.79	51.75	<0.001
Neutral	26.78	25.39	47.83		6.27	28.22	65.51		6.97	37.98	55.05	
Somewhat high / Very high	24.38	13.22	62.40		5.03	13.69	81.27		19.59	27.90	52.52	
**Patient behavior**												
Patient trust												

Very low / Somewhat low	25.29	10.10	64.61	<0.001	3.42	10.84	85.75	<0.001	13.89	24.65	61.45	<0.001
Neutral	26.07	17.91	56.02		7.28	19.04	73.68		18.52	31.82	49.67	
Somewhat high / Very high	24.45	24.18	51.37		11.02	25.34	63.64		30.49	33.52	35.99	
Unreasonable request from the patient												
Very low / Somewhat low	25.12	20.63	54.25	0.003	8.21	23.19	68.61	<0.001	28.53	30.53	40.94	<0.001
Neutral	24.05	16.24	59.71		6.09	16.62	77.29		14.35	34.88	50.77	
Somewhat high / Very high	27.04	9.53	63.43		4.08	9.43	86.49		11.33	22.12	66.55	

Table S8. Descriptive statistics for Organizational Behavior with Work Engagement of Physician

	Pride				Pleasure			
	Low	Neutral	High	*P* value	Low	Neutral	High	*P* value
**Organizational fairness**								
Pay equity				<0.001				<0.001
Very bad	61.33	28.95	9.72		68.98	22.85	8.17	
Somewhat bad	44.73	45.06	10.21		55.38	33.63	10.99	
Neutral	28.33	47.39	24.28		37.18	44.10	18.72	
Somewhat good/ Very good	18.68	41.63	39.69		25.39	31.64	42.97	
Task fairness				<0.001				<0.001
Very bad	70.47	22.00	7.54		73.93	21.38	4.68	
Somewhat bad	49.07	41.49	9.44		60.17	30.80	9.03	
Neutral	33.60	47.90	18.50		44.91	38.86	16.22	
Somewhat good/ Very good	24.71	37.07	38.22		27.69	36.84	35.47	
**Leadership attention**								
Interests attention				<0.001				<0.001
Very bad	60.12	31.14	8.74		68.86	22.79	8.35	
Somewhat bad	43.97	42.54	13.49		52.85	36.79	10.36	
Neutral	27.41	49.40	23.20		36.29	42.82	20.89	
Somewhat good/ Very good	25.08	38.31	36.61		34.01	32.99	32.99	
Opinions attention				<0.001				<0.001
Very bad	59.00	31.58	9.42		68.30	23.11	8.60	
Somewhat bad	42.62	42.75	14.63		52.29	36.77	10.94	
Neutral	27.85	51.21	20.93		36.20	44.90	18.90	
Somewhat good/ Very good	23.25	35.03	41.72		30.67	29.39	39.94	
**Team interaction**								
Number of dinners with colleagues				<0.001				<0.001
0-1 time	45.98	39.57	14.45		54.44	32.32	13.24	
2 times	31.46	47.19	21.35		40.90	42.02	17.08	
3 times	19.76	43.71	36.53		29.94	40.72	29.34	
≥4 times	29.00	41.99	29.00		39.57	33.48	26.96	
Number of clinical case meetings				<0.001				<0.001
0-1 time	49.80	39.71	10.48		55.70	33.42	10.88	
2 times	38.80	45.27	15.93		50.47	35.02	14.51	
3 times	38.21	42.11	19.69		46.78	36.65	16.57	
≥4 times	38.98	39.43	21.60		48.12	33.36	18.52	

Table S9. Descriptive statistics for Organizational Behavior with Work-home Conflicts of Physician

	Affecting care for family				Feeling guilty towards family				Receiving complaint from family			
	Low	Neutral	High	*P* value	Low	Neutral	High	*P* value	Low	Neutral	High	*P* value
**Organizational fairness**												
Pay equity				<0.001				<0.001				<0.001
Very bad	26.71	7.28	66.00		3.20	6.95	89.85		11.37	18.21	70.42	
Somewhat bad	26.64	14.54	58.82		5.77	15.98	78.25		17.20	30.08	52.72	
Neutral	22.41	20.39	57.20		6.58	21.08	72.34		19.24	35.86	44.89	
Somewhat good/ Very good	31.64	23.05	45.31		15.23	28.52	56.25		36.19	34.63	29.18	
Task fairness				0.009				<0.001				<0.001
Very bad	29.12	5.91	64.97		2.86	4.90	92.24		11.00	13.65	75.36	
Somewhat bad	27.36	10.60	62.03		5.58	13.30	81.12		13.73	26.47	59.80	
Neutral	23.43	18.25	58.32		6.54	18.50	74.97		18.18	34.20	47.63	
Somewhat good/ Very good	26.71	22.37	50.91		8.94	25.69	65.37		30.66	32.49	36.84	
**Leadership attention**												
Interests attention				<0.001				<0.001				<0.001
Very bad	23.65	9.32	67.03		4.13	9.72	86.15		14.44	20.43	65.13	
Somewhat bad	25.78	13.21	61.01		5.18	15.16	79.66		16.69	30.27	53.04	
Neutral	25.17	20.88	53.95		7.82	21.22	70.96		19.07	35.22	45.70	
Somewhat good/ Very good	32.20	20.00	47.80		8.81	23.05	68.14		27.12	33.56	39.32	
Opinions attention				<0.001				<0.001				<0.001
Very bad	23.57	10.26	66.17		3.79	10.71	85.50		16.45	20.06	63.49	
Somewhat bad	26.05	12.20	61.75		5.98	14.50	79.52		14.12	31.55	54.33	
Neutral	23.83	21.40	54.77		5.98	20.75	73.27		18.41	35.14	46.45	
Somewhat good/ Very good	35.35	20.70	43.95		14.38	25.56	60.06		29.07	34.82	36.10	
**Team interaction**												
Number of dinners with colleagues				<0.001				<0.001				<0.001
0-1 time	25.24	12.74	62.02		5.41	13.62	80.97		15.70	27.41	56.89	
2 times	25.84	23.82	50.34		7.43	24.55	68.02		23.60	35.51	40.90	
3 times	18.56	28.74	52.69		4.19	29.34	66.47		24.40	37.50	38.10	
≥4 times	31.74	16.52	51.74		10.87	19.57	69.57		23.81	29.87	46.32	
Number of clinical case meetings				<0.001				<0.001				0.170
0-1 time	29.62	17.17	53.21		8.14	19.69	72.18		18.61	30.67	50.72	
2 times	24.41	19.21	56.38		5.52	21.92	72.56		15.77	33.75	50.47	
3 times	25.00	16.80	58.20		4.69	15.43	79.88		15.59	30.99	53.41	
≥4 times	23.78	12.04	64.18		5.72	12.20	82.08		18.90	25.60	55.50	

Table S10. Multivariable Logistic Regression Results for Correlates of Work Engagement and Work-home Conflicts of Physician-Sensitivity Analysis

Organizational behavior	%	**Work engagement**				**Work-home Conflicts**					
		**High Pride**		**High Pleasure**		**High level of effect on care for family**		**High level of guilt towards family**		**High level of complaint from family**	
		**OR(95%CI)**	***P* value**	**OR(95%CI)**	***P* value**	**OR(95%CI)**	***P* value**	**OR(95%CI)**	***P* value**	**OR(95%CI)**	***P* value**
**Organizational fairness**											
Pay equity											
Very bad	27.88	1[reference]		1[reference]		1[reference]		1[reference]		1[reference]	
Somewhat bad	27.74	0.55(0.36-0.83)	0.005	0.84(0.56-1.28)	0.422	0.84(0.65-1.08)	0.182	0.66(0.47-0.94)	0.020	0.72(0.56-0.92)	0.010
Neutral	36.50	1.05(0.70-1.57)	0.818	0.93(0.61-1.42)	0.746	0.96(0.73-1.27)	0.785	0.66(0.45-0.95)	0.025	0.68(0.52-0.89)	0.005
Somewhat good/ Very good	7.89	0.75(0.43-1.33)	0.329	1.32(0.75-2.32)	0.329	0.65(0.43-1.00)	0.051	0.38(0.23-0.63)	<0.001	0.38(0.24-0.58)	<0.001
Task fairness											
Very bad	15.11	1[reference]		1[reference]		1[reference]		1[reference]		1[reference]	
Somewhat bad	21.51	1.01(0.60-1.69)	0.975	1.68(0.93-3.04)	0.085	1.01(0.75-1.37)	0.940	0.48(0.30-0.77)	0.002	0.63(0.46-0.87)	0.006
Neutral	49.93	1.27(0.78-2.07)	0.341	2.12(1.20-3.74)	0.009	1.03(0.77-1.39)	0.841	0.48(0.30-0.76)	0.002	0.48(0.35-0.66)	<0.001
Somewhat good/ Very good	13.46	2.37(1.35-4.18)	0.003	2.64(1.39-5.01)	0.003	0.98(0.67-1.44)	0.915	0.45(0.26-0.77)	0.004	0.52(0.35-0.77)	0.001
**Leadership attention**											
Interests attention											
Very bad	31.35	1[reference]		1[reference]		1[reference]		1[reference]		1[reference]	
Somewhat bad	23.76	1.01(0.64-1.59)	0.979	0.70(0.43-1.14)	0.153	0.76(0.56-1.01)	0.061	1.03(0.70-1.51)	0.891	0.97(0.72-1.30)	0.825
Neutral	35.82	1.22(0.77-1.92)	0.391	1.09(0.68-1.74)	0.735	0.77(0.57-1.05)	0.098	0.86(0.58-1.27)	0.444	0.87(0.64-1.18)	0.366
Somewhat good/ Very good	9.07	1.96(1.12-3.45)	0.019	1.23(0.69-2.21)	0.483	0.68(0.45-1.03)	0.066	1.18(0.70-1.99)	0.529	0.81(0.53-1.24)	0.330
Opinions attention											
Very bad	33.30	1[reference]		1[reference]		1[reference]		1[reference]		1[reference]	
Somewhat bad	24.18	1.07(0.70-1.63)	0.744	0.97(0.62-1.52)	0.885	1.18(0.89-1.56)	0.259	1.17(0.81-1.69)	0.407	0.97(0.73-1.28)	0.825
Neutral	32.88	1.11(0.71-1.74)	0.634	1.27(0.80-2.02)	0.318	0.87(0.65-1.18)	0.386	1.03(0.70-1.53)	0.868	1.05(0.78-1.43)	0.734
Somewhat good/ Very good	9.63	1.40(0.81-2.43)	0.224	1.86(1.07-3.26)	0.029	0.64(0.42-0.96)	0.032	0.73(0.45-1.20)	0.215	1.09(0.72-1.67)	0.681
**Team interaction**											
Number of dinners with colleagues											
0-1 time	74.04	1[reference]		1[reference]		1[reference]		1[reference]		1[reference]	
2 times	13.70	1.03(0.73-1.44)	0.878	0.80(0.56-1.14)	0.225	0.71(0.56-0.90)	0.004	0.62(0.47-0.82)	0.001	0.65(0.50-0.82)	<0.001

3 times	5.16	1.48(0.93-2.37)	0.102	1.27(0.80-2.02)	0.314	0.68(0.47-0.98)	0.037	0.50(0.33-0.74)	0.001	0.47(0.32-0.69)	<0.001
≥4 times	7.10	1.96(1.30-2.96)	0.001	2.11(1.39-3.19)	<0.001	0.71(0.52-0.97)	0.034	0.48(0.33-0.70)	<0.001	0.72(0.52-0.99)	0.044
Number of clinical case meetings											
0-1 time	23.57	1[reference]		1[reference]		1[reference]		1[reference]		1[reference]	
2 times	19.58	1.47(1.00-2.18)	0.052	1.30(0.88-1.90)	0.184	1.21(0.95-1.55)	0.128	1.12(0.84-1.50)	0.436	1.15(0.89-1.48)	0.287
3 times	15.82	1.73(1.16-2.59)	0.007	1.36(0.91-2.04)	0.130	1.35(1.04-1.76)	0.026	1.80(1.30-2.50)	<0.001	1.16(0.88-1.52)	0.282
≥4 times	41.03	1.91(1.36-2.69)	<0.001	1.42(1.02-1.99)	0.040	1.74(1.40-2.16)	<0.001	1.98(1.51-2.59)	<0.001	1.27(1.02-1.58)	0.035
**Socio-demographic characteristics**											
Sex											
Men	56.58	1[reference]		1[reference]		1[reference]		1[reference]		1[reference]	
Women	43.42	0.95(0.73-1.24)	0.729	0.94(0.72-1.22)	0.624	1.16(0.97-1.39)	0.100	1.08(0.87-1.35)	0.491	0.87(0.73-1.05)	0.150
Age, y											
≤34	29.02	1[reference]		1[reference]		1[reference]		1[reference]		1[reference]	
35-44	34.63	1.28(0.81-2.04)	0.290	1.08(0.69-1.71)	0.731	0.90(0.67-1.20)	0.477	0.85(0.59-1.23)	0.399	1.46(1.09-1.97)	0.012
≥45	36.35	1.74(1.03-2.94)	0.039	1.42(0.84-2.39)	0.192	0.84(0.59-1.19)	0.325	0.63(0.41-0.99)	0.043	1.56(1.09-2.24)	0.015
Marital status											
Single/other	16.48	1[reference]		1[reference]		1[reference]		1[reference]		1[reference]	
Married	83.52	1.16(0.77-1.75)	0.474	0.89(0.59-1.33)	0.562	1.01(0.78-1.32)	0.933	1.20(0.87-1.65)	0.273	1.01(0.77-1.33)	0.925
Education level											
Undergraduate and below	52.93	1[reference]		1[reference]		1[reference]		1[reference]		1[reference]	
Masters	35.61	0.89(0.67-1.17)	0.401	0.87(0.65-1.15)	0.323	1.16(0.96-1.40)	0.134	1.27(1.00-1.62)	0.049	1.48(1.22-1.81)	<0.001
PhD	11.47	0.85(0.58-1.26)	0.417	0.82(0.55-1.23)	0.340	1.27(0.95-1.70)	0.100	1.05(0.74-1.48)	0.797	1.22(0.92-1.64)	0.171
Economic status											
Very bad	12.96	1[reference]		1[reference]		1[reference]		1[reference]		1[reference]	
Somewhat bad	20.12	0.79(0.46-1.35)	0.390	1.22(0.68-2.17)	0.502	1.30(0.97-1.76)	0.083	0.89(0.57-1.37)	0.583	0.67(0.48-0.92)	0.013
Neutral	58.63	1.22(0.76-1.97)	0.409	1.65(0.97-2.82)	0.065	1.39(1.05-1.85)	0.022	0.72(0.48-1.09)	0.119	0.60(0.44-0.81)	0.001
Good	8.28	2.75(1.53-4.93)	0.001	2.99(1.58-5.66)	0.001	1.48(0.97-2.26)	0.066	0.65(0.38-1.11)	0.114	0.67(0.43-1.03)	0.070
Title											
Primary / other	24.22	1[reference]		1[reference]		1[reference]		1[reference]		1[reference]	
Middle	31.63	0.74(0.46-1.18)	0.205	0.84(0.53-1.34)	0.471	1.02(0.76-1.39)	0.875	1.37(0.94-2.00)	0.097	0.97(0.71-1.32)	0.854
High	44.15	1.20(0.70-2.05)	0.511	0.99(0.58-1.69)	0.967	1.17(0.81-1.69)	0.395	1.60(1.02-2.51)	0.039	0.93(0.64-1.35)	0.702
**Hospital and Departmental characteristics**											
Hospital level											

Secondary	15.06	1[reference]		1[reference]		1[reference]		1[reference]		1[reference]	
Tertiary	84.94	1.20(0.82-1.75)	0.345	0.92(0.64-1.32)	0.645	0.78(0.60-1.00)	0.052	1.12(0.83-1.53)	0.458	0.92(0.71-1.19)	0.533
Hospital type											
Traditional Chinese medicine	27.78	1[reference]		1[reference]		1[reference]		1[reference]		1[reference]	
Western medicine	72.22	0.91(0.70-1.20)	0.518	0.82(0.62-1.08)	0.149	1.02(0.84-1.23)	0.835	1.13(0.90-1.42)	0.299	1.01(0.84-1.23)	0.891
Academic status											
Nonteaching	80.32	1[reference]		1[reference]		1[reference]		1[reference]		1[reference]	
Teaching	19.68	1.00(0.73-1.36)	0.985	0.94(0.68-1.30)	0.717	1.01(0.81-1.27)	0.909	0.79()	0.087	0.96(0.77-1.21)	0.743
Physician specialty											
Internal medicine	52.94	1[reference]		1[reference]		1[reference]		1[reference]		1[reference]	
Surgery	47.06	1.35(1.04-1.74)	0.023	1.05(0.81-1.35)	0.737	0.87(0.73-1.04)	0.135	0.89(0.71-1.10)	0.277	1.08(0.90-1.29)	0.414
The ratio of physicians to beds											
<0.20	28.62	1[reference]		1[reference]		1[reference]		1[reference]		1[reference]	
0.20-0.30	40.05	1.27(0.95-1.70)	0.105	0.99(0.74-1.33)	0.942	0.91(0.75-1.12)	0.378	0.83(0.64-1.07)	0.151	0.89(0.72-1.09)	0.243
≥0.30	31.34	1.16(0.85-1.59)	0.356	1.12(0.82-1.54)	0.478	0.89(0.72-1.11)	0.303	0.70(0.54-0.92)	0.010	0.84(0.67-1.04)	0.109
**Family support**											
Very low / Somewhat low	3.52	1[reference]		1[reference]		1[reference]		1[reference]		1[reference]	
Neutral	17.66	0.64(0.30-1.36)	0.244	0.56(0.26-1.24)	0.155	1.27(0.79-2.04)	0.329	2.09(1.24-3.52)	0.006	1.50(0.92-2.46)	0.108
Somewhat high / Very high	78.82	1.08(0.54-2.15)	0.831	1.32(0.65-2.70)	0.447	2.32(1.48-3.62)	<0.001	4.94(3.01-8.11)	<0.001	1.18(0.74-1.87)	0.497
**Patient behavior**											
Patient trust											
Very low / Somewhat low	42.51	1[reference]		1[reference]		1[reference]		1[reference]		1[reference]	
Neutral	46.24	2.68(1.99-3.61)	<0.001	2.11(1.56-2.85)	<0.001	0.83(0.69-1.00)	0.048	0.71(0.57-0.90)	0.005	0.97(0.81-1.17)	0.742
Somewhat high / Very high	11.26	8.42(5.75-12.33)	<0.001	5.23(3.58-7.63)	<0.001	0.85(0.63-1.14)	0.276	0.69(0.49-0.98)	0.036	0.75(0.55-1.02)	0.063
Unreasonable request from the patient											
Very low / Somewhat low	32.22	1[reference]		1[reference]		1[reference]		1[reference]		1[reference]	
Neutral	33.89	0.70(0.54-0.91)	0.009	0.88(0.67-1.15)	0.359	1.25(1.02-1.52)	0.033	1.60(1.26-2.02)	<0.001	1.46(1.20-1.79)	<0.001
Somewhat high / Very high	33.89	0.47(0.34-0.66)	<0.001	0.51(0.36-0.71)	<0.001	1.29(1.04-1.60)	0.020	2.32(1.76-3.04)	<0.001	2.21(1.78-2.75)	<0.001

Table S11. Multivariable Logistic Regression Results for Correlates of Work Engagement or Work-home Conflicts of Physician-Sensitivity Analysis

Organizational behavior	%	**Work engagement**				**Work-home Conflicts**					
		**High Pride**		**High Pleasure**		**High level of effect on care for family**		**High level of guilt towards family**		**High level of complaint from family**	
		**OR(95%CI)**	***P* value**	**OR(95%CI)**	***P* value**	**OR(95%CI)**	***P* value**	**OR(95%CI)**	***P* value**	**OR(95%CI)**	***P* value**
**Organizational fairness**											
Pay equity											
Very bad	27.88	1[reference]		1[reference]		1[reference]		1[reference]		1[reference]	
Somewhat bad	27.74	0.55(0.36-0.83)	0.005	0.83(0.54-1.26)	0.374	0.84(0.65-1.08)	0.179	0.66(0.47-0.94)	0.020	0.72(0.56-0.92)	0.010
Neutral	36.50	1.05(0.70-1.57)	0.811	0.92(0.61-1.41)	0.713	0.97(0.74-1.28)	0.838	0.68(0.47-0.99)	0.042	0.68(0.52-0.90)	0.007
Somewhat good/ Very good	7.89	0.73(0.41-1.29)	0.272	1.23(0.70-2.18)	0.471	0.65(0.43-1.00)	0.051	0.39(0.23-0.65)	<0.001	0.39(0.25-0.60)	<0.001
Task fairness											
Very bad	15.11	1[reference]		1[reference]		1[reference]		1[reference]		1[reference]	
Somewhat bad	21.51	1.00(0.60-1.69)	0.991	1.61(0.89-2.92)	0.117	1.00(0.74-1.36)	0.989	0.50(0.31-0.80)	0.004	0.65(0.47-0.90)	0.010
Neutral	49.93	1.23(0.75-2.01)	0.410	1.94(1.10-3.43)	0.023	1.02(0.76-1.38)	0.876	0.50(0.32-0.80)	0.003	0.50(0.36-0.69)	<0.001
Somewhat good/ Very good	13.46	2.30(1.30-4.07)	0.004	2.40(1.25-4.58)	0.008	0.99(0.67-1.46)	0.950	0.49(0.28-0.84)	0.009	0.55(0.36-0.82)	0.003
**Leadership attention**											
Interests attention											
Very bad	31.35	1[reference]		1[reference]		1[reference]		1[reference]		1[reference]	
Somewhat bad	23.76	1.03(0.65-1.62)	0.912	0.71(0.43-1.15)	0.163	0.75(0.56-1.01)	0.059	1.03(0.70-1.51)	0.893	0.95(0.71-1.28)	0.755
Neutral	35.82	1.21(0.77-1.91)	0.413	1.07(0.66-1.73)	0.780	0.77(0.57-1.05)	0.098	0.87(0.59-1.30)	0.504	0.88(0.65-1.19)	0.407
Somewhat good/ Very good	9.07	1.94(1.10-3.42)	0.023	1.17(0.65-2.12)	0.597	0.68(0.45-1.02)	0.063	1.19(0.71-2.00)	0.515	0.82(0.54-1.25)	0.352
Opinions attention											
Very bad	33.30	1[reference]		1[reference]		1[reference]		1[reference]		1[reference]	
Somewhat bad	24.18	1.10(0.72-1.68)	0.662	1.00(0.63-1.57)	0.992	1.19(0.89-1.58)	0.237	1.22(0.84-1.77)	0.289	0.98(0.74-1.30)	0.881
Neutral	32.88	1.15(0.73-1.79)	0.553	1.33(0.83-2.13)	0.230	0.89(0.65-1.20)	0.438	1.11(0.75-1.64)	0.606	1.09(0.80-1.48)	0.572
Somewhat good/ Very good	9.63	1.44(0.83-2.51)	0.197	2.01(1.14-3.55)	0.016	0.64(0.43-0.97)	0.036	0.77(0.47-1.27)	0.308	1.15(0.75-1.75)	0.530
**Team interaction**											
Number of dinners with colleagues											
0-1 time	74.04	1[reference]		1[reference]		1[reference]		1[reference]		1[reference]	
2 times	13.70	1.01(0.72-1.42)	0.952	0.77(0.54-1.11)	0.161	0.71(0.56-0.90)	0.005	0.63(0.48-0.83)	0.001	0.65(0.51-0.83)	0.001
3 times	5.16	1.45(0.90-2.33)	0.123	1.20(0.75-1.92)	0.450	0.68(0.48-0.98)	0.040	0.52(0.35-0.77)	0.001	0.48(0.33-0.71)	<0.001

≥4 times	7.10	1.89(1.25-2.87)	0.003	2.03(1.33-3.09)	0.001	0.71(0.52-0.98)	0.036	0.50(0.34-0.72)	<0.001	0.75(0.54-1.04)	0.081
Number of clinical case meetings											
0-1 time	23.57	1[reference]		1[reference]		1[reference]		1[reference]		1[reference]	
2 times	19.58	1.51(1.02-2.24)	0.040	1.35(0.92-1.99)	0.129	1.20(0.94-1.54)	0.143	1.15(0.86-1.53)	0.357	1.17(0.90-1.51)	0.236
3 times	15.82	1.78(1.19-2.66)	0.005	1.42(0.94-2.13)	0.092	1.36(1.04-1.77)	0.025	1.89(1.36-2.63)	<0.001	1.19(0.90-1.56)	0.223
≥4 times	41.03	1.91(1.35-2.69)	<0.001	1.43(1.02-2.02)	0.040	1.74(1.39-2.16)	<0.001	2.06(1.57-2.70)	<0.001	1.29(1.04-1.62)	0.023
**Socio-demographic characteristics**											
Sex											
Men	56.58	1[reference]		1[reference]		1[reference]		1[reference]		1[reference]	
Women	43.42	0.93(0.71-1.22)	0.622	0.91(0.69-1.19)	0.480	1.16(0.97-1.39)	0.101	1.06(0.85-1.33)	0.588	0.86(0.72-1.04)	0.116
Age, y											
≤34	29.02	1[reference]		1[reference]		1[reference]		1[reference]		1[reference]	
35-44	34.63	1.34(0.84-2.15)	0.218	1.16(0.73-1.85)	0.517	0.89(0.67-1.20)	0.448	0.83(0.57-1.20)	0.322	1.45(1.08-1.95)	0.014
≥45	36.35	1.79(1.05-3.05)	0.032	1.49(0.88-2.54)	0.138	0.83(0.58-1.19)	0.309	0.63(0.40-0.98)	0.042	1.58(1.10-2.27)	0.013
Marital status											
Single/other	16.48	1[reference]		1[reference]		1[reference]		1[reference]		1[reference]	
Married	83.52	1.15(0.76-1.74)	0.498	0.88(0.58-1.33)	0.537	1.01(0.77-1.32)	0.943	1.17(0.85-1.62)	0.335	1.00(0.76-1.31)	0.998
Education level											
Undergraduate and below	52.93	1[reference]		1[reference]		1[reference]		1[reference]		1[reference]	
Masters	35.61	0.90(0.68-1.19)	0.468	0.90(0.67-1.20)	0.475	1.17(0.97-1.42)	0.105	1.30(1.02-1.66)	0.032	1.49(1.22-1.82)	<0.001
PhD	11.47	0.83(0.56-1.23)	0.351	0.81(0.54-1.22)	0.316	1.30(0.97-1.74)	0.078	1.08(0.76-1.53)	0.652	1.23(0.92-1.65)	0.160
Economic status											
Very bad	12.96	1[reference]		1[reference]		1[reference]		1[reference]		1[reference]	
Somewhat bad	20.12	0.75(0.44-1.29)	0.301	1.16(0.65-2.07)	0.624	1.32(0.98-1.79)	0.069	0.92(0.60-1.42)	0.711	0.68(0.49-0.94)	0.019
Neutral	58.63	1.15(0.71-1.86)	0.558	1.55(0.90-2.65)	0.114	1.40(1.05-1.86)	0.021	0.76(0.50-1.15)	0.196	0.62(0.46-0.84)	0.002
Good	8.28	2.62(1.45-4.72)	0.001	2.85(1.50-5.43)	0.001	1.48(0.97-2.27)	0.068	0.70(0.41-1.21)	0.202	0.72(0.46-1.12)	0.149
Title											
Primary / other	24.22	1[reference]		1[reference]		1[reference]		1[reference]		1[reference]	
Middle	31.63	0.74(0.46-1.20)	0.222	0.85(0.53-1.35)	0.482	1.02(0.75-1.38)	0.889	1.36(0.93-1.99)	0.111	0.96(0.70-1.31)	0.790
High	44.15	1.19(0.69-2.06)	0.523	0.99(0.57-1.71)	0.974	1.18(0.82-1.71)	0.366	1.66(1.05-2.62)	0.029	0.94(0.65-1.36)	0.728
**Hospital and Departmental characteristics**											
Hospital level											
Secondary	15.06	1[reference]		1[reference]		1[reference]		1[reference]		1[reference]	
Tertiary	84.94	1.23(0.84-1.79)	0.294	0.95(0.66-1.38)	0.801	0.77(0.59-0.99)	0.043	1.11(0.81-1.51)	0.519	0.91(0.70-1.19)	0.503

Hospital type											
Traditional Chinese medicine	27.78	1[reference]		1[reference]		1[reference]		1[reference]		1[reference]	
Western medicine	72.22	0.90(0.68-1.18)	0.431	0.79(0.60-1.05)	0.100	1.02(0.84-1.23)	0.834	1.11(0.88-1.40)	0.363	1.00(0.82-1.21)	0.990
Academic status											
Nonteaching	80.32	1[reference]		1[reference]		1[reference]		1[reference]		1[reference]	
Teaching	19.68	0.98(0.72-1.35)	0.923	0.93(0.68-1.29)	0.680	1.02(0.81-1.28)	0.871	0.80(0.61-1.04)	0.099	0.97(0.77-1.21)	0.773
Physician specialty											
Internal medicine	52.94	1[reference]		1[reference]		1[reference]		1[reference]		1[reference]	
Surgery	47.06	1.37(1.06-1.78)	0.017	1.09(0.84-1.41)	0.539	0.87(0.73-1.04)	0.131	0.90(0.72-1.12)	0.333	1.09(0.91-1.30)	0.374
The ratio of physicians to beds											
<0.20	28.62	1[reference]		1[reference]		1[reference]		1[reference]		1[reference]	
0.20-0.30	40.05	1.24(0.93-1.67)	0.144	0.96(0.71-1.29)	0.786	0.91(0.75-1.12)	0.372	0.83(0.64-1.07)	0.148	0.88(0.72-1.08)	0.235
≥0.30	31.34	1.11(0.81-1.53)	0.516	1.08(0.78-1.48)	0.646	0.89(0.71-1.10)	0.287	0.69(0.53-0.91)	0.008	0.84(0.67-1.04)	0.114
**Family support**											
Very low / Somewhat low	3.52	1[reference]		1[reference]		1[reference]		1[reference]		1[reference]	
Neutral	17.66	0.83(0.38-1.83)	0.651	0.76(0.33-1.73)	0.516	1.28(0.80-2.07)	0.304	2.18(1.29-3.68)	0.004	1.51(0.92-2.48)	0.103
Somewhat high / Very high	78.82	1.31(0.63-2.72)	0.463	1.58(0.74-3.37)	0.234	2.34(1.49-3.66)	<0.001	5.41(3.27-8.95)	<0.001	1.23(0.77-1.96)	0.390
**Patient behavior**											
Patient trust											
Very low / Somewhat low	42.51	1[reference]		1[reference]		1[reference]		1[reference]		1[reference]	
Neutral	46.24	2.68(1.99-3.61)	<0.001	2.11(1.56-2.86)	<0.001	0.83(0.69-1.01)	0.060	0.79(0.62-1.00)	0.052	1.03(0.85-1.24)	0.785
Somewhat high / Very high	11.26	8.42(5.74-12.36)	<0.001	5.20(3.55-7.63)	<0.001	0.85(0.62-1.16)	0.294	0.79(0.55-1.13)	0.203	0.83(0.60-1.15)	0.267
Unreasonable request from the patient											
Very low / Somewhat low	32.22	1[reference]		1[reference]		1[reference]		1[reference]		1[reference]	
Neutral	33.89	0.75(0.57-0.98)	0.036	0.98(0.74-1.29)	0.896	1.25(1.02-1.53)	0.030	1.62(1.28-2.05)	<0.001	1.47(1.20-1.80)	<0.001
Somewhat high / Very high	33.89	0.50(0.36-0.70)	<0.001	0.57(0.40-0.80)	0.001	1.28(1.03-1.59)	0.024	2.23(1.69-2.94)	<0.001	2.15(1.73-2.68)	<0.001
**Work-home Conflicts**											
Affecting care for family											
Very low / Somewhat low		1[reference]		1[reference]							
Neutral		1.24(0.81-1.89)	0.324	0.98(0.64-1.51)	0.937						
Somewhat high / Very high		1.20(0.88-1.64)	0.256	1.17(0.85-1.60)	0.336						
Feeling guilty towards family											
Very low / Somewhat low											

Neutral	0.64(0.37-1.10)	0.105	0.91(0.53-1.57)	0.748						
Somewhat high / Very high	0.73(0.44-1.23)	0.241	0.94(0.56-1.59)	0.823						
Receiving complaint from family										
Very low / Somewhat low	1[reference]		1[reference]							
Neutral	0.56(0.40-0.80)	0.001	0.54(0.38-0.75)	<0.001						
Somewhat high / Very high	0.62(0.44-0.88)	0.007	0.45(0.32-0.64)	<0.001						
**Work engagement**										
Pride										
Very low / Somewhat low					1[reference]		1[reference]		1[reference]	
Neutral					1.16(0.94-1.44)	0.170	0.89(0.68-1.18)	0.421	0.92(0.74-1.14)	0.446
Somewhat high / Very high					1.14(0.83-1.57)	0.421	0.91(0.62-1.34)	0.627	1.03(0.75-1.44)	0.837
Pleasure										
Very low / Somewhat low					1[reference]		1[reference]		1[reference]	
Neutral					0.79(0.64-0.97)	0.025	0.66(0.51-0.85)	0.002	0.86(0.70-1.06)	0.168
Somewhat high / Very high					0.86(0.63-1.17)	0.334	0.66(0.46-0.95)	0.024	0.61(0.44-0.83)	0.002
